# Patency of Arterial Versus Venous Grafts in Multisegmental Coronary Artery Disease

**DOI:** 10.3390/jcm15187254

**Published:** 2026-09-18

**Authors:** Yoganiranjana Dharuman, Egan Kalmykov, Mirko Doss

**Affiliations:** 1 Department of Cardiothoracic Surgery, University Hospital Aachen, Pauwelsstraße 30, 52074 Aachen, Germany; 2 Department of Endovascular Surgery, University Clinic of Brandenburg/Havel, 14770 Brandenburg, Germany; 3 Department of Cardiothoracic Surgery, Helios Hospital Siegburg, 53721 Siegburg, Germany

**Keywords:** coronary artery bypass grafting, vascular patency, arterial graft, saphenous vein graft, radial artery, multisegmental (diffuse) coronary artery disease

## Abstract

**Background:** Arterial grafts are widely believed to offer superior long-term patency compared with saphenous vein grafts, yet in multisegmental coronary artery disease the diseased runoff bed may attenuate this advantage. In this study, we compared the long-term patency of arterial and venous conduits in patients revascularised for multisegmental coronary artery disease. **Methods:** We retrospectively analysed 467 bypass grafts (100 arterial internal thoracic artery grafts, 351 saphenous vein grafts and 16 radial artery grafts) in 133 patients with multisegmental coronary artery disease treated by coronary artery bypass grafting, including long anastomoses where required. Graft patency was assessed by multidetector computed tomography or coronary angiography. Patency over time was estimated by the Kaplan–Meier method and compared with the log-rank test. Because imaging was symptom-driven rather than protocol-based, patency estimates apply to the imaged subgroup. **Results:** Early patency was comparably high for arterial (97%) and venous (96%) conduits. Patency curves diverged progressively: at 12 years, estimated patency was 83% for arterial grafts versus 65% for venous grafts (*p* < 0.01). Radial artery grafts maintained 87% patency over the available follow-up. **Conclusions:** These findings suggest that the long-term patency advantage of arterial conduits may be preserved in patients with multisegmental coronary artery disease, including where long anastomoses are required, and warrant confirmation in larger cohorts with systematic imaging follow-up.

## 1. Introduction

The choice of bypass conduit is one of the principal determinants of long-term outcome after coronary artery bypass grafting. The superiority of the left internal thoracic artery (ITA) over the saphenous vein for grafting of the left anterior descending artery is well established, and preferential use of arterial conduits has been associated with improved survival and reduced cardiac events [1]. Contemporary meta-analytic data further support multiple arterial grafting, which has been associated with a significant long-term survival benefit over single arterial grafting [2]. Nevertheless, the saphenous vein graft (SVG) remains the most frequently used conduit worldwide because of its availability and versatility, even though its long-term patency is inferior to that of arterial grafts [3,4]. Contemporary imaging-based analyses confirm that graft failure remains frequent and is independently associated with adverse clinical events [5], and current European consensus recommendations accordingly emphasise optimised conduit selection and management to improve graft durability [6]. Current European guidelines reflect this evidence, recommending the left internal thoracic artery to the left anterior descending artery as the standard of care and endorsing the use of a second arterial conduit in appropriately selected patients to improve long-term outcome [7].

The durability advantage of arterial conduits has a well-defined biological basis. The internal thoracic artery is an elastic, largely non-fenestrated artery whose endothelium retains vigorous vasomotor and antithrombotic function; it expresses higher levels of endothelial nitric oxide synthase and releases more nitric oxide than the saphenous vein, thereby limiting platelet aggregation and smooth-muscle proliferation and rendering the conduit comparatively resistant to intimal hyperplasia and graft atherosclerosis [8]. The saphenous vein, by contrast, is abruptly exposed to arterial pressure and pulsatile flow after implantation, and its failure is a multifactorial process comprising early thrombosis, medial and intimal hyperplasia, and late accelerated atherosclerosis [9]. These structural and functional differences provide a mechanistic rationale for the progressive, time-dependent divergence in patency that is repeatedly observed between arterial and venous grafts, and they motivate the preferential use of arterial conduits wherever the target vessel and conduit quality permit.

Multisegmental, or diffuse, coronary artery disease poses a particular challenge to durable revascularisation. When atherosclerosis involves long segments of the target vessel, especially the distal graftable portions, conventional short anastomoses onto a diseased bed may provide inadequate runoff and predispose to early graft failure [9]. In these circumstances techniques such as coronary endarterectomy, sequential grafting, surgical patch angioplasty and long arteriotomies have been used to achieve complete revascularisation [10,11]. The diffusely diseased runoff bed, however, may itself influence graft fate, and it is uncertain whether the patency advantage of arterial over venous conduits observed in conventional disease is preserved in the multisegmental setting. Long-segment (extended) anastomosis, in which the conduit is laid over an extended arteriotomy without removal of the underlying plaque, has emerged as a further option that preserves the native lumen and side branches while avoiding the additional risk associated with endarterectomy, and it has been reported to achieve favourable long-term anastomotic remodelling [12].

Much of the evidence underpinning conduit selection derives from patients with focal, discretely graftable lesions, and randomised trials of graft patency have generally excluded or under-represented diffusely diseased vessels. As a consequence, the behaviour of arterial and venous grafts anastomosed to a diffusely diseased runoff bed, particularly when long anastomoses are required, has not been well characterised, and it is biologically plausible that a compromised distal bed, with its higher outflow resistance and reduced runoff, could attenuate the intrinsic advantage of arterial conduits. Addressing this uncertainty requires robust, imaging-based follow-up, since both multidetector computed tomography and invasive coronary angiography provide accurate and highly concordant assessment of graft occlusion [13].

What remains unestablished is whether the arterial patency advantage, demonstrated largely in focal disease, is preserved specifically when grafts are placed onto diffusely diseased, multi-segmental vessels requiring long anastomoses; the present study was designed to address this gap.

The purpose of this study was to compare the long-term patency of arterial and venous grafts in patients revascularised for multisegmental coronary artery disease requiring long anastomoses. We hypothesised that arterial conduits would retain a durable patency advantage over saphenous vein grafts despite the diseased target bed. As secondary observations, we examined the pattern and timing of any divergence in patency between conduit classes, the territorial distribution of graft failure, and the early behaviour of radial artery grafts, together with perioperative morbidity and mortality, in order to place the patency findings in their clinical context.

## 2. Methods

We retrospectively analysed patients with multisegmental coronary artery disease who underwent coronary artery bypass grafting at our institution. A total of 133 consecutive patients met the inclusion criteria and CABG procedures were performed between 2013 and 2014. A total of 467 bypass grafts were available for patency analysis and were classified by conduit type into three groups: 100 arterial grafts (internal thoracic artery), 351 saphenous vein grafts, and 16 radial artery grafts. All patients had triple-vessel disease and received at least one graft onto a diffusely diseased vessel, in addition to conventional grafts. None had undergone previous coronary artery bypass surgery. Baseline clinical characteristics and risk factors are summarised in Table 1.

Each patient received at least one long (extended) anastomosis onto a diffusely diseased vessel, together with conventional and, where appropriate, sequential grafts; the 467 grafts analysed therefore comprise all grafts placed (both long-anastomosis and conventional).

### Study Design and Setting

This single-centre observational study was conducted at a tertiary cardiac surgical unit. Consecutive patients with angiographically documented triple-vessel disease and multisegmental involvement of at least one target vessel, who underwent isolated coronary artery bypass grafting and received at least one graft onto a diffusely diseased vessel, were eligible for inclusion. Patients who had undergone previous coronary artery bypass surgery, and those undergoing concomitant valve or aortic procedures, were excluded so that graft patency could be attributed to the index operation. Conduit selection followed contemporary guideline-directed practice, with preferential use of the internal thoracic artery and, where anatomically feasible, a second arterial conduit [7]. For the patency analysis, grafts were classified into three groups according to conduit type: internal thoracic artery, saphenous vein, and radial artery.

For the purposes of this study, multisegmental disease denoted a coronary vessel with continuous or near-continuous atherosclerotic involvement, most marked along its distal, graftable course, in which a short, focal anastomosis could not be expected to restore adequate distal perfusion. A graft was classified as a long anastomosis when the coronary opening spanned the plaque-bearing segment over more than 1.5 cm, as opposed to the roughly 0.7 cm openings used for standard anastomoses. This 1.5 cm value is a pragmatic operative threshold distinguishing an extended arteriotomy from a standard focal anastomosis; no universally accepted numerical cut-off exists. The definitions were prespecified and applied consistently by the same surgical team. These definitions were applied at operation on the basis of the pre-operative angiogram together with direct intraoperative inspection and palpation of the vessel, so that the distinction between a standard and a long anastomosis was anatomical rather than arbitrary, reflecting the length of the coronary artery over which the arteriotomy had to traverse a plaque-bearing wall in order to reach an adequate distal lumen. Anastomosis length was measured intraoperatively along the arteriotomy by the same surgical team.

**Operative technique.** All procedures were performed for isolated coronary artery disease under cardiopulmonary bypass using cold blood cardioplegic arrest at moderate systemic hypothermia (32 °C). Conduits consisted of the internal thoracic artery, radial artery, and saphenous vein, as summarised in Table 2.

For the patency analysis, each distal anastomosis of a sequential graft was counted separately.

When a long coronary anastomosis was indicated, the coronary arteriotomy was initiated in a relatively preserved segment adjacent to the diseased vessel and then extended longitudinally through the stenotic portion while carefully maintaining the true lumen. Importantly, the atherosclerotic plaque was not removed, and the diseased segment was deliberately left without endarterectomy. The selected conduit was opened longitudinally to match the length of the coronary arteriotomy and was subsequently anastomosed using a continuous running suture along the entire extent of the incision. The deliberate decision to leave the atherosclerotic plaque in situ was intended to preserve the native intima and side branches and to avoid the added thrombogenicity and remodelling disturbance associated with removal of the atherosclerotic core, an approach consistent with reports of favourable long-term remodelling after long-segment reconstruction [12]. In addition to the long anastomoses, conventional and, where appropriate, sequential grafts were constructed to achieve complete revascularisation of all significantly diseased territories [10,11].

Postoperatively, all patients received subcutaneous heparin for one week in combination with aspirin (100 mg once daily). Thereafter, patients were maintained on lifelong aspirin unless a specific contraindication or an alternative antithrombotic indication was present, and guideline-directed secondary-prevention pharmacotherapy, including statins, was prescribed [7].

Dual antiplatelet therapy (aspirin plus clopidogrel for six months) was administered only to patients who had presented with an acute coronary syndrome (ST-elevation or non-ST-elevation myocardial infarction, or unstable angina), in accordance with guideline recommendations; oral anticoagulation was used only when separately indicated.

**Follow-up and patency assessment.** The mean follow-up period was 5 years, with imaging follow-up extending to 12 years. Graft evaluation was not undertaken as part of a systematic study protocol. Instead, imaging was performed on a clinical basis when patients presented with symptoms during the postoperative course, and graft patency was assessed by multidetector computed tomography or coronary angiography according to the clinical indication. With either modality, a graft was scored as occluded whenever contrast opacification of the target coronary artery beyond the anastomosis could not be seen. Patency was recorded separately for each conduit type and target territory. Both multidetector computed tomography and invasive coronary angiography are established and highly concordant modalities for the detection of graft occlusion, with reported sensitivity and specificity exceeding 95% [13], and the choice between them was governed by the clinical question and local availability. A graft demonstrating antegrade opacification of the distal target vessel was classified as patent, whereas absence of distal opacification defined occlusion; grafts were not graded for non-occlusive stenosis. For each occluded graft, the date of the first imaging study demonstrating occlusion was taken as the event time.

Because imaging was clinically driven rather than serial, the true time of occlusion lies between the last patent assessment and that study; event times are therefore interval-censored and occlusion timing is approximate. Early patency denotes patency assessed at the first available postoperative imaging study.

**Clinical follow-up.** Clinical follow-up was obtained from outpatient records and referring cardiologists. Freedom from angina, myocardial infarction, reoperation and reintervention were Kaplan–Meier estimates; reoperation denotes any repeat cardiac surgical procedure and reintervention denotes any repeat coronary revascularisation (surgical or percutaneous). Patients lost to follow-up were censored at the date last known to be event-free.

**Statistical analysis.** Continuous variables are expressed as mean ± standard deviation. Graft patency over time was estimated by the Kaplan–Meier method and compared between conduit groups with the log-rank test. A *p*-value < 0.05 was considered statistically significant. Data were compiled and analysed using Microsoft Access and Microsoft Excel (Microsoft Corp., Redmond, WA, USA) and IBM SPSS Statistics version 28 (IBM Corp., Armonk, NY, USA). Categorical variables are presented as counts and percentages. Graft survival, defined as freedom from occlusion, was analysed from the date of operation to the date of the imaging study documenting occlusion; grafts shown to be patent at the last available imaging study were censored at that time. Kaplan–Meier estimates were generated separately for the arterial, venous, and radial conduits, and differences between curves were assessed with the log-rank test. Because the imaging follow-up was retrospective and symptom-driven, the number of grafts at risk at the latest time points was small, and the corresponding patency estimates are reported with this limitation in mind.

## 3. Results

**Perioperative outcome.** There were no intraoperative deaths. The average aortic cross-clamp time was 72.2 min (range 26–167 min) and the average cardiopulmonary bypass time was 111.7 min (range 37–229 min). One patient required intra-aortic balloon pump support for low cardiac output and four required re-exploration for haemorrhage. Early mortality was 5.7%. Causes of early and late death are shown in Table 3. At five years, freedom from angina was 83.9%, freedom from myocardial infarction 96.8%, freedom from reoperation 100% and freedom from reintervention 92.5%.

**Figure 1:**
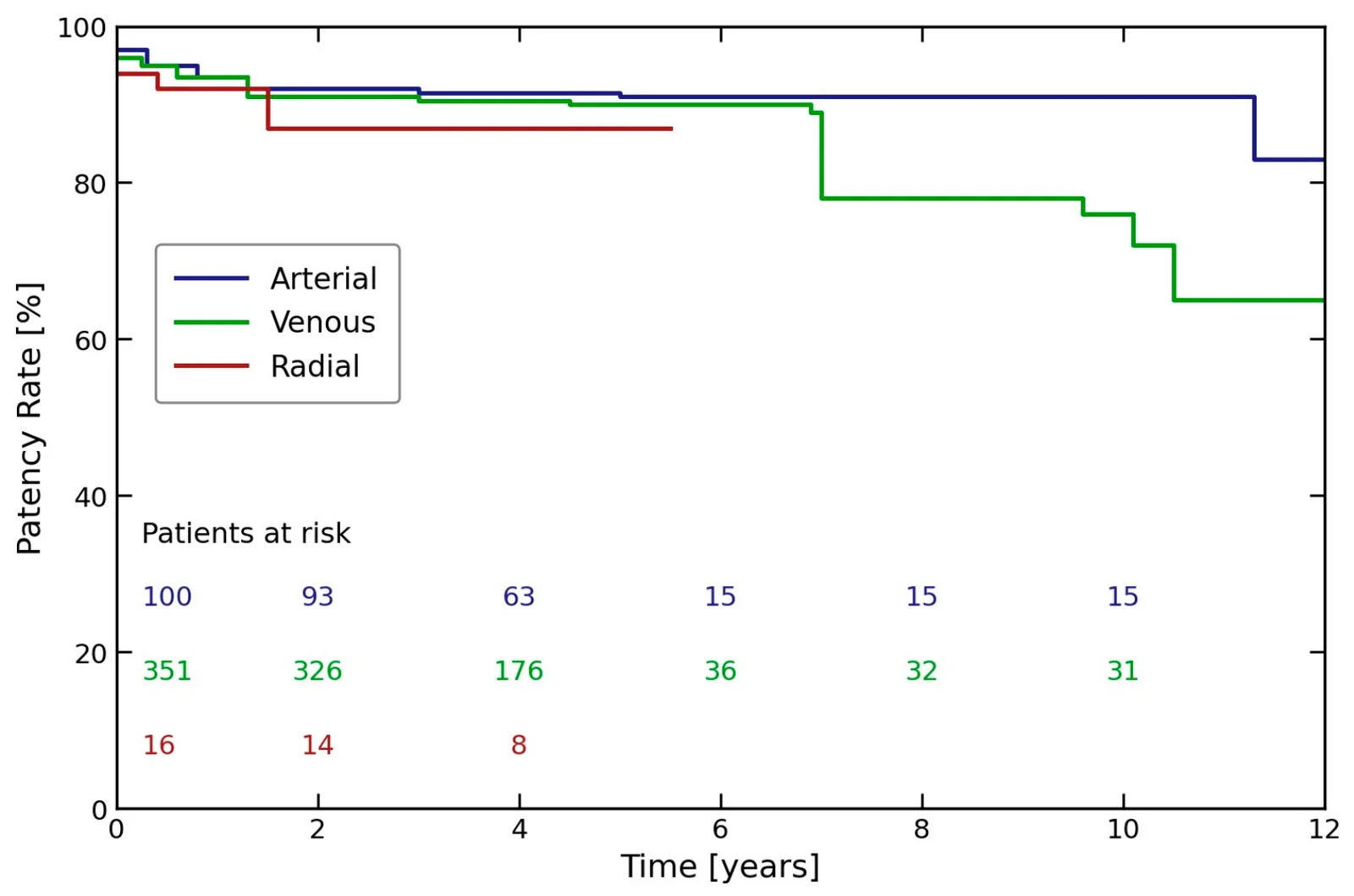
Kaplan–Meier estimates of graft patency by conduit type, arterial (ITA), venous (SVG) and radial, with the numbers of grafts at risk shown beneath the curves.

**Graft patency: arterial versus venous.** Early patency was comparably high for both conduit classes (arterial 97%, venous 96%). Thereafter, the Kaplan–Meier curves diverged progressively in favour of the arterial grafts (Figure 1). Arterial patency remained above 90% through the first decade and was estimated at 83% at 12 years, whereas venous patency declined more steeply after the fifth year, reaching approximately 65% at 12 years; the difference was statistically significant (log-rank *p* < 0.01). The patency estimates for each conduit at successive time points are detailed in Table 4.

**Radial artery grafts.** The 16 radial artery grafts maintained a patency of approximately 87% over the available follow-up (to about 5.5 years). Given the small number of radial grafts and the shorter follow-up, these results are reported as exploratory and descriptive only; no formal comparison with the other conduits is intended (Figure 1).

## 4. Discussion

In this observational cohort, arterial grafting was associated with durable long-term patency advantage over saphenous vein grafts. Although early patency was comparable between conduit classes, the Kaplan–Meier curves diverged progressively, and by 12 years arterial patency (83%) substantially exceeded venous patency (65%). This pattern mirrors the conduit hierarchy established in conventional coronary disease and indicates that the diffusely diseased target bed does not abolish the intrinsic advantage of arterial conduits.

The superiority of the internal thoracic artery is one of the most consistent observations in coronary surgery. Loop and colleagues first demonstrated that internal thoracic artery grafting to the left anterior descending artery improved 10-year survival and reduced late cardiac events compared with vein grafting [1]. Angiographic follow-up studies have repeatedly confirmed that arterial grafts occlude far less frequently than vein grafts over time: Fitzgibbon and colleagues, in a 25-year angiographic series, reported progressive vein graft attrition, whereas internal thoracic artery grafts remained largely patent [3], and the Department of Veterans Affairs cooperative study documented markedly higher long-term patency for internal mammary than for saphenous vein grafts [4]. Sabik and colleagues showed that the patency advantage of the internal thoracic artery is present across coronary systems [14]. Our 12-year results are fully consistent with this body of evidence.

Saphenous vein graft failure is a multifactorial process encompassing early thrombosis, intimal hyperplasia and late accelerated atherosclerosis, and remains the principal limitation of vein-based revascularisation [9]. In the multisegmental setting these mechanisms may be compounded by impaired distal runoff, and it is notable that in our series the greatest venous attrition occurred in the circumflex territory, consistent with the higher failure rates reported for vein grafts onto smaller, diffusely diseased targets [15]. The comparatively preserved arterial patency, even when arterial conduits were used for long anastomoses, supports preferential arterial grafting whenever technically feasible.

The role of the radial artery as a second arterial conduit has been clarified by randomised evidence. The RADIAL pooled analysis demonstrated superior patency and improved clinical outcomes for radial artery compared with saphenous vein grafts [16], and the RAPS trial similarly reported lower angiographic occlusion for radial than vein grafts [17]. The ART trial extended the rationale for multiple arterial grafting, although the survival benefit of bilateral internal thoracic artery grafting was not confirmed at 10 years in the intention-to-treat analysis [18]. In our cohort the small number of radial grafts precludes firm inference, but their patency tracked with the arterial rather than the venous conduits, in keeping with these trials.

Achieving complete revascularisation is important in diffuse disease, as incomplete revascularisation is associated with worse outcomes [19]. Where the target vessel is diffusely diseased, several strategies have been proposed. Coronary endarterectomy achieves revascularisation of otherwise ungraftable vessels but has been associated with reduced graft patency in several series and meta-analyses [10,20], and contemporary series of endarterectomy and patch angioplasty for diffuse disease report comparable heterogeneity in graft patency [11,21]. Against this background, the long anastomosis offers a technically straightforward alternative that preserves the native lumen and avoids endarterectomy, and in our experience arterial conduits used in this fashion retained excellent long-term patency.

The clinical implications of conduit choice extend beyond angiographic patency. Complete arterial revascularisation and internal thoracic artery use have been linked to improved long-term survival and freedom from reintervention [1,22,23], and sequential vein grafting has been associated with graft failure in some analyses [15]. The durable arterial patency observed here, together with acceptable perioperative mortality, reinforces the case for maximising arterial grafting in patients with multisegmental disease, reserving vein grafts for territories where arterial conduit length or quality is insufficient. Cross-clamp times in our series (mean 72 min) were comparable to those reported for conventional grafting and for long-segment coronary reconstruction, and such reconstructions have been performed without excess perioperative myocardial injury, indicating that arterial grafting of diffusely diseased vessels with long anastomoses does not impose a disproportionate ischaemic penalty [12]. The early mortality of 5.7% should be interpreted in the context of the high-risk profile of this cohort: all patients had triple-vessel, multisegmental disease requiring long-segment reconstruction, in whom higher perioperative risk is expected. Any preference for arterial conduits must be weighed against practical and patient-specific considerations, including conduit availability and quality, target-vessel size and run-off, the risk of competitive flow, patient age and comorbidity, and, for bilateral internal thoracic artery use, the risk of sternal wound complications. The radial artery remains a well-established second arterial conduit, and conduit choice should be individualised rather than applied uniformly.

## 5. Conclusions

In patients revascularised for multisegmental coronary artery disease, arterial grafts retained a durable long-term patency advantage over saphenous vein grafts despite the diffusely diseased target bed. Early patency was comparable between conduit classes, but the curves diverged progressively, with an arterial patency of 83% versus a venous patency of 65% at 12 years. These findings support the preferential use of arterial conduits, including for long anastomoses, whenever technically feasible, with saphenous vein grafts reserved for territories not amenable to arterial grafting. These findings suggest that the long-term patency advantage of arterial conduits may be preserved in patients with multisegmental coronary artery disease, including where long anastomoses are required, and warrant confirmation in larger cohorts with systematic, protocol-driven imaging follow-up.

### Limitations

This study has several limitations. It is a retrospective, single-centre analysis without randomisation, and conduit allocation was determined by the operating surgeon, introducing potential selection bias. Importantly, graft imaging was not performed systematically but only when patients developed symptoms during follow-up; this symptom-driven assessment introduces a referral (selection) bias, since asymptomatic patients with patent grafts were less likely to be imaged, and the reported patency figures must therefore be interpreted with caution. In addition, the number of grafts under observation at the latest time points was small, particularly for the arterial and radial groups, so the 10- and 12-year estimates are correspondingly uncertain. Because imaging was not serial, occlusion timing is interval-censored and approximate. Graft failure was defined as complete occlusion only; grafts were not graded for non-occlusive stenosis, which may underestimate graft disease. Finally, the analysis focused on graft patency; clinical endpoints such as survival and repeat revascularisation were not the primary outcome and warrant dedicated study. Future studies should ideally incorporate systematic, protocol-driven angiographic or computed-tomographic follow-up rather than symptom-driven imaging, include larger radial-artery cohorts with longer follow-up, link graft patency to hard clinical endpoints such as survival and repeat revascularisation, and directly compare the long anastomosis with coronary endarterectomy in diffusely diseased target vessels.

## Figures and Tables

**Table 1:** Patient baseline characteristics.

Characteristic	Value
Male/female	98/35 (74%/26%)
Mean age	67 ± 6 years
Triple-vessel disease	133 (100%)
Arterial hypertension	74 (55.4%)
Diabetes mellitus	38 (28.3%)
Hyperlipidaemia	39 (29.5%)
Current/former smoking (combined)	28 (21.1%)
Previous CABG	0 (0%)

**Table 2:** Operative data and procedural details. Times are mean (range).

Variable	Value	Range
Aortic cross-clamp time	72.2 min	26–167 min
Cardiopulmonary bypass time	111.7 min	37–229 min
Arterial (ITA) grafts	100	—
Saphenous vein grafts	351	—
Radial artery grafts	16	—
Long anastomoses	≥1 per patient	1.5–3.5 cm
IABP support	1 patient	—

**Table 3:** Causes of early (30-day) and late (5-year) mortality.

Cause	Early (n)	Late (n)
Ventricular fibrillation	3	—
Cardiac tamponade	1	—
Left ventricular failure	2	3
Myocardial infarction	—	2
Stroke	—	2
Total	6 (5.7%)	7 (6.7%)

**Table 4:** Long-term graft patency by conduit type. Arterial grafts show superior late patency compared with saphenous vein grafts (log-rank *p* < 0.01).

Conduit	n Grafts	Early	5-Year	10-Year	12-Year
Arterial (ITA)	100	97%	92%	90%	83%
Venous (SVG)	351	96%	89%	70%	65%
Radial artery	16	94%	87%	n/a *	n/a *

ITA: internal thoracic artery; SVG: saphenous vein graft. * Radial follow-up limited to approximately 5.5 years.

## Data Availability

The data presented in this study are available on request from the corresponding author.

## Abbreviations

CABG	coronary artery bypass grafting
CAD	coronary artery disease
CPB	cardiopulmonary bypass
CT	computed tomography
CX	circumflex artery
IABP	intra-aortic balloon pump
ITA	internal thoracic artery
LAD	left anterior descending artery
MD-CT	multidetector computed tomography
RCA	right coronary artery
SVG	saphenous vein graft
